# NGF-β and BDNF levels are altered in male patients with chronic schizophrenia: effects on clinical symptoms

**DOI:** 10.1186/s12888-025-06685-8

**Published:** 2025-03-13

**Authors:** Haidong Yang, Qing Tian, Lingshu Luan, Man Yang, Chuanwei Li, Xiaobin Zhang

**Affiliations:** 1 Department of Psychiatry, The Fourth People’s Hospital of Lianyungang, The Affiliated KangDa College of Nanjing Medical University, Lianyungang, 222003 P.R. China; 2 Suzhou Psychiatric Hospital, Institute of Mental Health, The Affiliated Guangji Hospital of Soochow University, Suzhou, 215137 P.R. China; 3 Xuzhou Medical University, Xuzhou, 221004 P.R. China

**Keywords:** Schizophrenia, NGF-β, BDNF, 1,25(OH)₂D, Clinical symptoms, Cognitive functions

## Abstract

**Background:**

Schizophrenia, a severe mental disorder with complex pathophysiology, involves neurotrophic factors, which play crucial roles in neurodevelopment and neuroplasticity. This study investigated NGF-β and BDNF levels in chronic schizophrenia and their association with clinical symptoms, cognitive function, and 1,25(OH)₂D levels.

**Methods:**

In this cross-sectional study, 72 male patients with chronic schizophrenia and 70 matched healthy controls were enrolled. Psychopathological symptoms were assessed using the Positive and Negative Syndrome Scale (PANSS), and cognitive function was evaluated using the Repeatable Battery for the Assessment of Neuropsychological Status (RBANS). The serum levels of NGF-β, BDNF, and 1,25(OH)₂D were measured.

**Results:**

Serum levels of NGF-β (F = 35.239, *P* < 0.001) and BDNF (F = 12.669, *P* < 0.001) were significantly decreased in patients with chronic schizophrenia compared to healthy controls. NGF-β levels were negatively correlated with PANSS negative symptoms (beta = -0.205, *t* = -2.098, *P* = 0.040) and positively correlated with 1,25(OH)₂D levels (*r* = 0.324, *P* = 0.006). Decreased serum BDNF concentrations were negatively correlated with language deficits (beta = -0.301, *t* = -2.762, *P* = 0.007). Significant associations were observed between chronic schizophrenia and reduced levels of NGF-β (B = 1.040, *P* < 0.001, RR = 2.829, 95% CI: 2.101−3.811) and BDNF (B = 0.526, *P* = 0.001, RR = 1.692, 95% CI: 1.241−2.306).

**Conclusions:**

Our findings indicated that NGF-β and BDNF levels were altered in chronic schizophrenia and were associated with clinical symptoms and vitamin D metabolism. These results provided new insight into the etiology of schizophrenia.

## Introduction

Schizophrenia is a heterogeneous, debilitating, and severe psychiatric disorder affecting approximately 1% of the global population [1, 2]. This condition exerts a particularly profound impact on male patients, with studies indicating that male often experience more severe symptoms, earlier onset, and poorer prognosis than female [3, 4]. Individuals with chronic schizophrenia typically face long-term cognitive impairments, social difficulties, and diminished quality of life. This substantially burdens the patient, their family, and society [5, 6]. Notably, male patients are more susceptible to persistent negative symptoms and cognitive deficits, factors that significantly compromise their social functioning and occupational capabilities [7, 8]. While the etiology of schizophrenia remains elusive, mounting evidence suggests that neurotrophic factors and oxidative stress play crucial roles in its pathophysiology [9, 10].

Nerve growth factor-β (NGF-β) is a crucial neurotrophic factor that plays a vital role in neuronal survival, growth, and differentiation [11, 12]. Recent studies have implicated NGF-β in the pathophysiology of schizophrenia [13]. Multiple investigations have reported abnormal NGF-β levels in schizophrenia patients, with these alterations correlating with the severity of psychopathological symptoms [12]. A meta-analysis revealed significantly reduced NGF-β levels in first-episode drug naïve schizophrenia patients [14]. Furthermore, a 3-year longitudinal study demonstrated an association between NGF-β levels and negative symptoms in first-episode patients [15]. Interestingly, Vasic et al. found a correlation between cerebrospinal fluid NGF-β levels and positive symptoms in patients with schizophrenia [16]. However, the correlation of NGF-β in chronic schizophrenia with psychopathological symptoms requires further investigation.

**Fig. 1:**
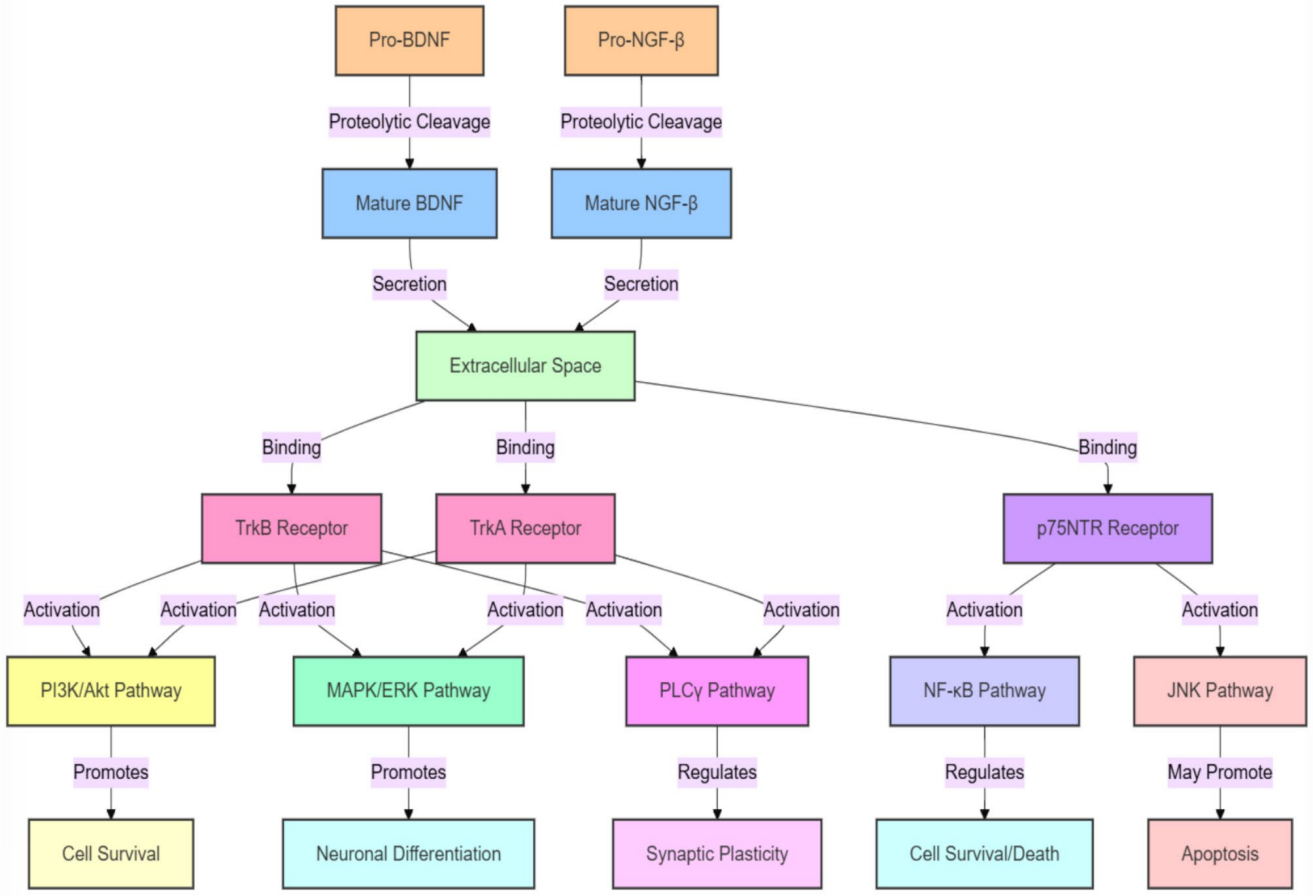
This diagram illustrates the signaling pathways of Brain-Derived Neurotrophic Factor (BDNF) and Nerve Growth Factor-β (NGF-β). Starting from their precursor proteins (Pro-BDNF and Pro-NGF-β), these factors undergo proteolytic cleavage to form mature neurotrophins, which are then secreted into the extracellular space. They bind to different receptors (TrkB, TrkA, and p75NTR), activating multiple signaling cascades (including PI3K/Akt, MAPK/ERK, PLCγ, NF-κB, and JNK pathways). These pathways ultimately regulate crucial biological processes such as cell survival, neuronal differentiation, synaptic plasticity, and apoptosis. In schizophrenia, dysregulation of these neurotrophic factors may lead to abnormal neurodevelopment, synaptic dysfunction, and altered neuroplasticity, affecting cognitive functions and emotional regulation, thus contributing to the pathophysiological mechanisms of the disorder

Brain-derived neurotrophic factor (BDNF) is another crucial neurotrophic factor that plays a pivotal role in neuroplasticity and cognitive function [17, 18]. Extensive research has demonstrated that alterations in BDNF levels are associated with the risk of schizophrenia onset, symptom severity, and cognitive impairments [19, 20]. A comprehensive meta-analysis encompassing 2,667 patients and 2,580 controls revealed significantly lower peripheral BDNF levels in individuals with schizophrenia compared to healthy controls [21]. This reduction was more pronounced in chronic patients and negatively correlated with disease duration. Another study found that the BDNF genetic polymorphism rs6265 (G/A) increased susceptibility to schizophrenia [22], and BDNF Val66Met genetic polymorphism was associated with some aspects of cognitive function, such as language performance [23]. In animal models, decreased BDNF expression led to schizophrenia-like behavioral changes [24], including reduced social interaction and cognitive dysfunction [25]. The NRG-β and BDNF pathway flow diagram is shown in Fig. 1.

Vitamin D is an important neurosteroid hormone that has garnered increasing attention in psychiatric research in recent years [26]. In the body, vitamin D exists primarily in two forms: 25-hydroxyvitamin D [25(OH)D] and 1,25-dihydroxyvitamin D [1,25(OH)₂D], with the latter being the biologically active form [27]. Beyond its classical role in calcium and phosphate metabolism regulation, 1,25(OH)₂D exhibits significant antioxidant and neuroprotective properties [28]. Studies have demonstrated that 1,25(OH)₂D effectively mitigates oxidative stress by enhancing antioxidant enzyme activity and reducing free radical production, thereby protecting neurons from oxidative damage [29]. Moreover, evidence suggests that 1,25(OH)₂D may influence neurological function by modulating the expression of neurotrophic factors, such as NGF [30]. A systematic review and meta-analysis revealed that patients with schizophrenia were more susceptible to vitamin D deficiency compared to healthy controls, and neonatal vitamin D deficiency was associated with an elevated risk of developing schizophrenia [31].

Based on this background, we hypothesized that alterations in NGF-β, BDNF, and vitamin D levels may be associated with symptom severity, cognitive deficits, and disease susceptibility. Furthermore, we postulated potential interactions between vitamin D and these neurotrophic factors. Therefore, the main objectives of this study were to (1) compare serum levels of NGF-β, BDNF, and 1,25(OH)₂D between male patients with chronic schizophrenia and healthy controls; (2) investigate the relationships among NGF-β, BDNF, and 1,25(OH)₂D levels and psychopathological symptoms and cognitive deficits; (3) explore potential associations among NGF-β, BDNF, and 1,25(OH)₂D; and (4) evaluate NGF-β, BDNF, and 1,25(OH)₂D levels as potential risk factors for chronic schizophrenia.

## Methods and subjects

### Subjects

This cross-sectional study was conducted in October 2020. The study population comprised two groups: patients with schizophrenia and healthy controls. The schizophrenia group consisted of long-term inpatients from the Psychiatric Department of Fourth People’s Hospital of Lianyungang. Hospitalization decisions were made following comprehensive clinical evaluations, which considered symptom severity, medication compliance, social functioning, and necessity for a structured environment and rehabilitation programs. The inclusion criteria for this group were the following: (1) a diagnosis of schizophrenia according to DSM-IV criteria; (2) male sex; (3) Han Chinese ethnicity; (4) age between 18 and 65 years; (5) hospitalization exceeding 2 years; (6) stable medication regimen for at least 12 months; (7) no anti-inflammatory treatment in the 4 weeks prior to enrollment; and (8) an education level sufficient to complete cognitive function tests.

The healthy control group was recruited from the community during the same period. These individuals did not meet DSM-IV Axis I diagnostic criteria and had no family history of psychiatric disorders. All participants completed a semi-structured questionnaire to collect information on age, gender, education, smoking history, body mass index (BMI), and, where applicable, duration of illness and age of onset. The exclusion criteria applied to both groups and included comorbid severe physical illnesses, neurodegenerative disorders, endocrine system diseases, alcohol or drug dependence, and the use of vitamin D supplements within the three months preceding enrollment. Each participant’s health status was determined through physical examination and laboratory tests, including complete blood count, liver and kidney function tests, blood glucose, and thyroid function assessments. All participants or their legal guardians provided written informed consent. The study protocol was approved by the Ethics Committee of Fourth People’s Hospital of Lianyungang.

### Clinical and cognitive assessment

Two senior psychiatrists with extensive experience assessed each participant’s clinical symptoms and cognitive function. The severity of clinical symptoms was evaluated using the Positive and Negative Syndrome Scale (PANSS). To ensure assessment reliability, inter-rater correlation coefficients were maintained above 0.8 for all scored items [32]. According to a previous study, PANSS total scores are categorized as follows: mild symptoms (< 58), moderate symptoms (58–74), and marked symptoms (≥75) [33]. Antipsychotic medication dosages were converted to chlorpromazine equivalents for standardized comparison [34]. Cognitive function was assessed using the Repeatable Battery for the Assessment of Neuropsychological Status (RBANS) [35]. The RBANS is a comprehensive neuropsychological instrument that evaluates five cognitive domains: immediate memory, visuospatial/constructional, language, attention, and delayed memory. Each domain consists of several subtests. Raw scores from these subtests were calculated and then converted to index scores, allowing for standardized comparisons across individuals. It is noteworthy that the reliability and validity of the RBANS have been validated in Chinese populations through multiple studies [36, 37].

### Measurement of serum NGF-β, BDNF, and 1,25(OH)₂D levels

Peripheral venous blood samples were collected from all participants between 07:00 and 09:00 AM following overnight fasting. Blood was drawn into anticoagulant-free tubes and centrifuged at 3,000 rpm for 15 min at 4 °C. The resulting serum samples were immediately aliquoted and stored at -80 °C until analysis. To ensure objectivity, serum samples were analyzed by technicians blinded to the clinical information. Serum levels of NGF-β, BDNF, and 1,25(OH)₂D were measured using the Luminex liquid suspension chip detection method, strictly adhering to the manufacturer’s protocol (R&D Systems, Minneapolis, MN, USA). This method demonstrated high precision, with intra-assay coefficients of variation (CV) ranging from 2.0 to 5.6% and inter-assay CV ranging from 3.7 to 6.1%.

### Statistical analysis

The Kolmogorov-Smirnov test was employed to assess data normality. Non-normally distributed data were transformed using natural logarithms to achieve normal distribution. Normally distributed continuous variables were compared using independent samples *t*-test or Student’s *t*-test, while non-normally distributed variables were analyzed using the Mann-Whitney U test. Categorical variables were compared using the chi-square test. Analysis of covariance (ANCOVA) was performed to determine group differences while adjusting for confounding factors such as education and BMI. Pearson’s correlation analysis was employed with stepwise multiple regression to control for confounding factors. NGF-β, BDNF, and 1,25(OH)₂D levels of all participants were dichotomized based on median values, coded as 0 (above median) and 1 (below median). Furthermore, a modified Poisson regression was used to identify predictors of chronic schizophrenia susceptibility, with diagnosis as the dependent variable and NGF-β, BDNF, and 1,25(OH)₂D (dichotomized), age, education, BMI, and smoking as independent variables. Statistical analyses were conducted using SPSS version 22.0 for Windows (IBM, USA). The sample size was calculated using G*Power 3.1. Cohen’s d-value was calculated to express effect size, with 0.2 indicating a small effect size, 0.5 a medium effect size, and 0.8 a large effect size. Statistical significance was set at *P* < 0.05.

## Results

### Sociodemographic and clinical characteristics

Regarding medication profiles, none of the participants were administered antidepressants, mood stabilizers, or somatic treatments during the study period. Eight patients (11.1%) were prescribed benzodiazepines on an as-needed for sleep management. For antipsychotic treatment, 40 patients (55.56%) underwent monotherapy, whereas 32 patients (44.44%) received combination therapy. Risperidone and quetiapine were the most commonly prescribed medications, administered to 12 patients (16.67%) and 8 patients (11.11%), respectively. A total of 26 patients (36.11%) were treated with clozapine-containing regimens, including 5 patients (6.94%) undergoing clozapine monotherapy and 21 patients (29.17%) on clozapine combination therapy. The detailed antipsychotic prescriptions included aripiprazole (*n* = 6, 8.33%), risperidone (*n* = 12, 16.67%), clozapine (*n* = 5, 6.94%), amisulpride (*n* = 4, 5.56%), olanzapine (*n* = 5, 6.94%), quetiapine (*n* = 8, 11.11%), clozapine-aripiprazole combination (*n* = 5, 6.94%), clozapine-risperidone combination (*n* = 7, 9.72%), clozapine-perphenazine combination (*n* = 10, 13.89%), clozapine-quetiapine combination (*n* = 4, 5.56%), and olanzapine-aripiprazole combination (*n* = 6, 8.33%). The sociodemographic and clinical characteristics of the male patients with chronic schizophrenia and healthy controls are shown in Table 1. The patients’ age range was 21 − 61 years. There was no significant difference in age or smoking between the patients and healthy controls (all *P* > 0.05). Significant differences were observed in education (*t* = -2.294, *P* = 0.022), BMI (*t* = -2.018, *P* = 0.046), and the RBANS total score and subscores (all, *P* < 0.05). The PANSS total scores ranged from 34 to 91 (mean: 58.76). Among the participants, 12 individuals (16.67%) scored ≥ 75, 26 individuals (36.11%) scored between 58 and 74, and 34 individuals (47.22%) scored < 58.

**Table 1:** Demographic and clinical characteristics of the male patients with chronic schizophrenia and the healthy controls (HC)

	Patients (*n* = 72)	HC (*n* = 70)	t/Z/χ2	*P*
Age (years)	40.83 ± 9.65	39.81 ± 9.47	0.635a	0.526
Education (years)	9.0 (6.0, 9.0)	9.0 (8.25, 12.0)	-2.294b	0.022
BMI (kg/m2)	24.38 ± 3.43	25.48 ± 3.05	-2.018a	0.046
Smoking (yes/no)	37/35	25/45	3.545c	0.060
Age of onset (years)	26.86 ± 8.33d	-	-	-
Duration of illness (years)	13.37 ± 8.65d	-	-	-
PANSS total score	58.76 ± 15.13d	-	-	-
P subscores	11.21 ± 4.71d	-	-	-
*N* subscores	29.32 ± 6.66d	-	-	-
G subscores	17.74 ± 7.21d	-	-	-
Equivalent dose of chlorpromazine (mg/d)	584.87 ± 190.35d	-	-	-
RBANS total score	58.51 ± 11.07	89.16 ± 11.70	-16.037a	< 0.001
Immediate memory	50.42 ± 17.26	84.27 ± 16.74	-11.862a	< 0.001
Visuospatial constructional	69.47 ± 14.62	90.69 ± 14.48	-8.685a	< 0.001
Language	71.51 ± 14.45	97.46 ± 10.12	-12.361a	< 0.001
Attention	82.33 ± 14.01	107.37 ± 12.87	-11.081a	< 0.001
Delay memory	55.33 ± 16.68	84.53 ± 17.32	-10.231a	< 0.001

BMI, body mass index; PANSS, Positive and Negative Syndrome Scale; RBANS, repeatable battery for the assessment of neuropsychological status; a, independent samples *t*-test; b, Mann-Whitney U test; c, χ2 test; d, Student’s *t*-test

### Serum NGF-β, BDNF, and 1,25(OH)₂D levels between patients and healthy controls

The median (interquartile range, IQR) of 1,25(OH)₂D (pmol/L) in the control group was 159.89, ranging from 96.28 to 528.28, with 25th and 75th percentiles of 132.79, 209.67, respectively. In the patient group, the median (IQR) was 154.04, ranging from 102.91 to 319.0, with 25th, and 75th percentiles of 126.73, 183.42, respectively.

**Fig. 2:**
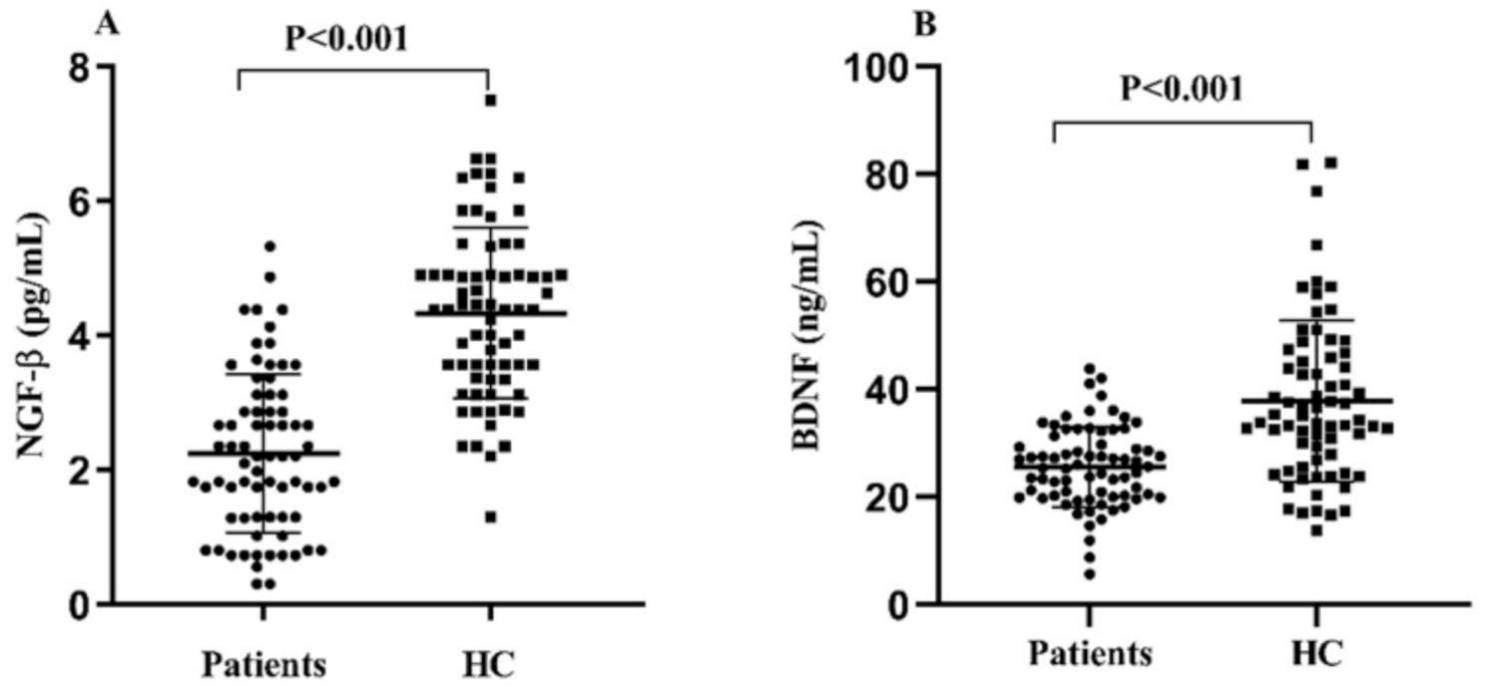
Serum NGF-β (**A**) and BDNF (**B**) levels in male patients with chronic schizophrenia compared with healthy controls (HC)

As shown in Table 2, there were significant differences in serum NGF-β (t = -10.145, *P* < 0.001), BDNF (*t* = -6.172, *P* < 0.001), and 1,25(OH)₂D (t = -2.265, *P* = 0.025) levels between patients and healthy controls. After adjusting for potential confounding factors, including education and BMI using ANCOVA, the differences in serum NGF-β (F = 35.239, *P* < 0.001, Fig. 2A) and BDNF (F = 12.669, *P* < 0.001, Fig. 2B) remained significant, whereas the difference in 1,25(OH)₂D levels (F = 2.148, *P* = 0.097, Fig. 2C) was no longer statistically significant.

**Table 2:** Serum NGF-β, BDNF, and 1,25(OH)₂D levels between patients and healthy controls (HC)

	Patients (*n* = 72)	HC (*n* = 70)	t	*P*
NGF-β (pg/mL)	2.25 ± 1.18	4.34 ± 1.27	-10.145	< 0.001
BDNF (ng/mL)	25.65 ± 7.43	37.86 ± 15.0	-6.172	< 0.001
1,25(OH)₂D (pmol/L)◊	2.19 ± 0.10	2.24 ± 0.17	-2.265	0.025

◊ results of natural logarithm transformation

### Association of serum NGF-β, BDNF, and 1,25(OH)₂D levels with psychopathology in patients

**Fig. 3:**
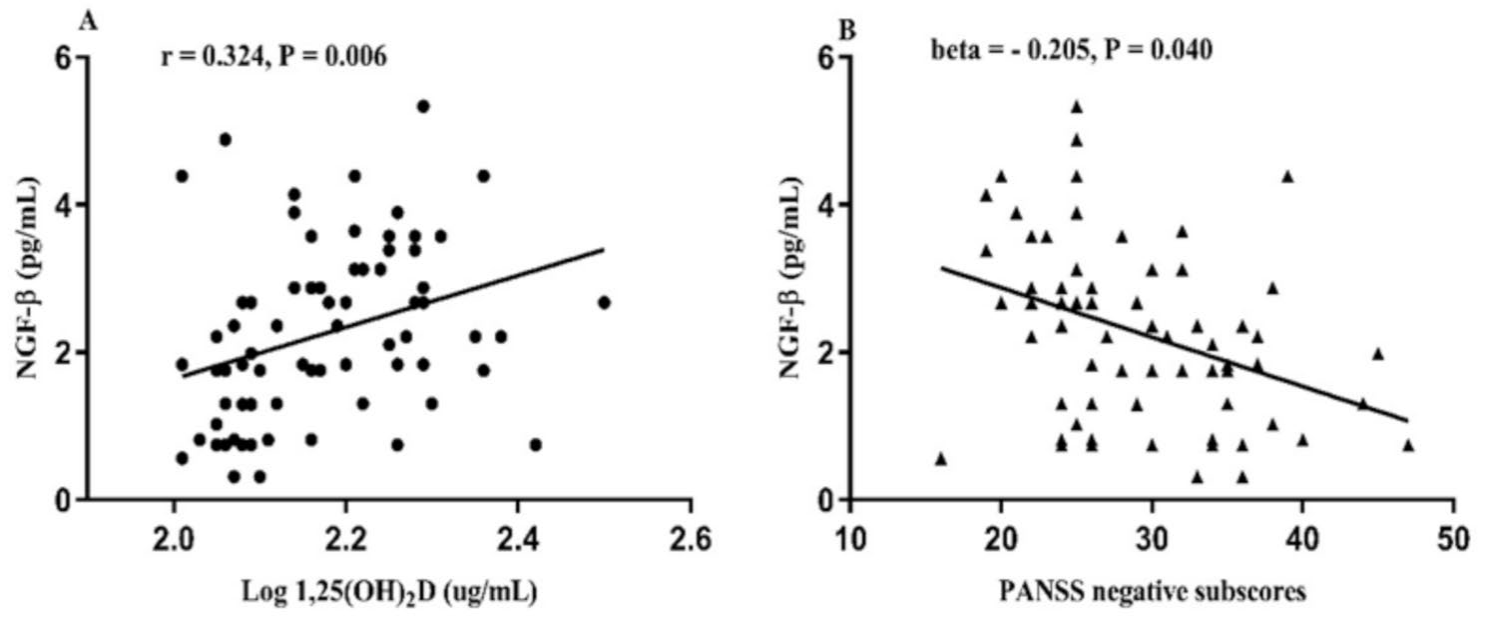
Relationship between serum NGF-β levels and 1,25(OH)₂D (**A**) and PANSS negative subscores (**B**) among patients

Pearson’s correlation analyses showed that serum NGF-β levels were negatively correlated with PANSS negative symptoms (*r* = -0.377, *P* = 0.001), general psychopathological symptoms (*r* = -0.270, *P* = 0.022), total score (*r* = -0.270, *P* = 0.022) and equivalent dose of chlorpromazine (*r* = -0.236, *P* = 0.046). However, serum NGF-β levels were positively correlated with 1,25(OH)₂D (*r* = 0.324, *P* = 0.006, Fig. 3A). Serum 1,25(OH)₂D levels were negatively associated with age (*r* = -0.265, *P* = 0.024). There was no correlation between BDNF or 1,25(OH)₂D levels and the PANSS total score and subscores (all, *P* > 0.05).

Further, after controlling for confounders of age, BMI, smoking, equivalent dose of chlorpromazine, age of onset, and duration of illness, the stepwise multiple regression revealed that PANSS negative subscores were negatively correlated with NGF-β (B = -1.156, beta = -0.205, *t* = -2.098, *P* = 0.040, Fig. 3B), and that equivalent doses of chlorpromazine (B = 0.018, *t* = 5.484, *P* < 0.001) and smoking (B = -2.734, *t* = -2.174, *P* = 0.033) were confounding factors.

### Association of serum NGF-β, BDNF, and 1,25(OH)₂D levels with cognitive function in patients and healthy controls

**Fig. 4:**
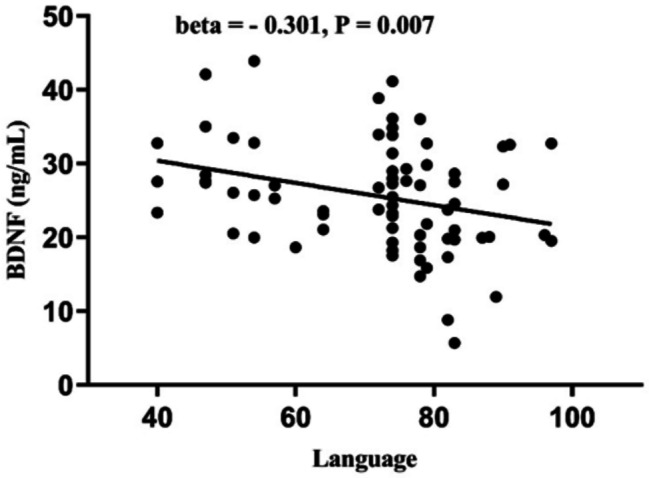
Correlation between BDNF levels and language among patients

Pearson’s correlation analyses demonstrated that BDNF was negatively associated with language (*r* = -0.293, *P* = 0.013) in male patients with chronic schizophrenia. However, no correlation was observed between NGF-β and 1,25(OH)₂D with the RBANS total score and subscores (all, *P* > 0.05). Furthermore, stepwise multiple regression indicated that language was negatively correlated with BDNF levels (B = -0.585, beta = -0.301, *t* = -2.762, *P* = 0.007, Fig. 4) and education (B = 1.526, beta = 0.311, *t* = 2.858, *P* = 0.006) after controlling for age, BMI, age of onset, duration of illness, smoking, and chlorpromazine equivalent dose. No association of NGF, BDNF, and 1,25(OH)₂D with RBANS total score and subscores was observed in the healthy controls (all, *P* > 0.05).

### Independent variables predicting susceptibility to chronic schizophrenia

To analyze independent variables predicting susceptibility to chronic schizophrenia, we categorized all participants’ NGF-β, BDNF, and 1,25(OH)₂D levels into low (coded as 1) and high (coded as 0) groups based on median values, while controlling for age, BMI, education, and smoking. Using diagnosis as the dependent variable, modified Poisson regression analysis showed that decreased NGF-β (B = 1.040, *P* < 0.001, RR = 2.829, 95% CI: 2.101–3.811) and BDNF (B = 0.526, *P* = 0.001, RR = 1.692, 95% CI: 1.241–2.306) levels were positively associated with the risk of chronic schizophrenia. However, no significant correlation between 1,25(OH)₂D and chronic schizophrenia was found (*P* = 0.526).

## Discussion

The main findings of this study are as follows: (1) serum levels of NGF-β and BDNF were significantly decreased in male patients with chronic schizophrenia compared to healthy controls; (2) the NGF-β levels were negatively correlated with PANSS negative symptoms and positively correlated with 1,25(OH)₂D levels; (3) decreased serum BDNF concentrations were negatively correlated with language deficits; and (4) the levels of NGF-β and BDNF may be associated with potential disease risk in chronic schizophrenia. To our knowledge, this is the first study to show a correlation between decreased serum NGF-β levels and psychopathological symptoms among Chinese male patients with chronic schizophrenia.

Our study revealed significantly reduced serum levels of NGF-β and BDNF in patients with chronic schizophrenia compared to healthy controls. This finding aligned with several previous investigations. A meta-analysis by Çakici et al. corroborated the substantial decrease in peripheral NGF-β and BDNF levels among schizophrenia patients [38]. Cecerska-Heryć et al. further demonstrated that BDNF reduction was present in both drug-naïve first-episode patients and chronic patients, correlating with disease duration [9]. However, another study on NGF levels yielded some discrepancies. Parikh et al. observed higher plasma NGF levels in chronic schizophrenia patients compared to drug-naïve first-episode patients, but both were lower than in healthy controls [39]. This variance might reflect the influence of disease stage and pharmacological intervention. Although our study did not establish a direct relationship between NGF and antipsychotic treatment, the potential impact of long-term therapy on NGF levels cannot be dismissed.

Moreover, Mei et al. indicated an association between BDNF levels and pharmacological treatment, offering a potential perspective on BDNF alterations in chronic patients [40]. We postulate that in the early stages of schizophrenia, NGF and BDNF are primarily linked to neurodevelopment, while in the chronic phase, their levels may be modulated by antipsychotic medication [39, 41]. The observed reduction in NGF-β and BDNF levels may indicate diminished neurotrophic support in schizophrenia patients. This deficiency in neurotrophic factors could lead to decreased neuroplasticity, subsequently affecting neuronal survival, growth, and function [18, 42, 43]. Nevertheless, the precise mechanisms require further investigation, particularly considering the chronic progression of the disease.

Our study revealed a negative correlation between reduced NGF-β levels and PANSS negative symptoms, suggesting a potential role for NGF-β in the negative symptomatology of schizophrenia. This finding aligned with research by Melkersson [44] and Malashenkova et al. [45], who reported a similar negative relationship between NGF levels and negative symptoms in schizophrenia patients. Su et al. further demonstrated an association between NGF gene variants and PANSS general psychopathology scores [46]. However, studies by Turkmen et al. [47] and Ermakov et al. [48] failed to establish significant correlations between NGF-β and psychopathological symptoms. These discrepancies may be attributed to variations in sample size, disease stage, or antipsychotic medication regimens [49]. The association between decreased NGF-β levels and exacerbation of negative symptoms in schizophrenia may reflect the crucial role of neurotrophic factors in the pathophysiology of the disorder, particularly in the development of negative symptoms.

Our study revealed that serum BDNF levels were negatively correlated exclusively with the language domain and not with other cognitive domains, suggesting a specific role of BDNF in language function in patients with schizophrenia. This finding aligned with the review by Nieto et al. [50], which reported significant associations between BDNF levels and multiple dimensions of cognitive deficits in schizophrenia patients. Isayeva et al. corroborated the positive correlation between BDNF and semantic fluency [51]. In animal studies, Schmidt et al. used the T-maze paradigm to demonstrate cognitive deficits in BDNF knockout mice [52]. As a key neurotrophic factor, BDNF is involved in neuroplasticity and synaptic function regulation. Previous studies showed that BDNF influences various cognitive processes, including learning, memory, and executive function, by modulating neuronal activity in the hippocampus and prefrontal cortex [53, 54, 55]. The lack of significant correlations between BDNF, NGF-β, vitamin D, and other cognitive domains could be attributed to the complex interplay of various factors influencing cognitive function in schizophrenia, such as disease severity, medication status, and educational background. BDNF, NGF-β, and vitamin D might influence distinct aspects of cognitive function via specific neurobiological pathways, and these effects could vary significantly among individuals [56, 57].

Although the initial analysis indicated lower 1,25(OH)₂D levels in patients with schizophrenia compared to healthy controls, this difference was no longer statistically significant after adjusting for confounding factors such as education level and BMI. This finding contrasts with previous research by Albiñana et al., who reported significantly lower vitamin D levels in schizophrenia patients [58]. Additionally, Adamson et al. found that vitamin D levels were significantly associated with BMI, age, gender, smoking, and illness duration [59]. Previous study has demonstrated a significant association between vitamin D deficiency and schizophrenia [31]. However, this association should be interpreted with caution, as vitamin D levels can be influenced by multiple factors in patients with schizophrenia, including lifestyle modifications (such as reduced outdoor activities and sun exposure), altered dietary habits, use of antipsychotic medications, and overall health status, all of which may impact circulating vitamin D levels.

Our study revealed a positive correlation between reduced NGF-β levels and 1,25(OH)₂D levels, suggesting a potential link between vitamin D and neurotrophic factors. The active form of vitamin D, 1,25(OH)₂D, possesses antioxidant properties [28, 29]. The vitamin D receptor is widely expressed in both neurons and glial cells, indicating a potential role in neural function [60]. In schizophrenia, the expression and function of NGF-β, a crucial neurotrophic factor, may be influenced by oxidative stress [61]. Previous research has indicated that oxidative stress might contribute to the pathogenesis of schizophrenia by affecting the expression and function of neurotrophic factors [62, 63, 64]. Vitamin D may influence NGF-β through various mechanisms, such as direct transcriptional regulation [30] and modulating oxidative stress levels [65, 66]. Gezen-Ak et al. demonstrated in a rat model that vitamin D affects NGF release in hippocampal neurons [67]. These findings collectively support the significance of the complex interplay among vitamin D, oxidative stress, and neurotrophic factors in the pathophysiology of schizophrenia, suggesting vitamin D as an important modulator of neurotrophic support within the central nervous system.

Our findings demonstrated decreased NGF-β and BDNF levels in chronic schizophrenia patients, suggesting their potential association with the risk of the disorder. Previous investigations have demonstrated significantly reduced BDNF and NGF levels in patients with chronic schizophrenia compared to healthy controls [9, 38], with NGF levels correlating with symptom severity [45, 46]. These neurotrophic factors are pivotal in maintaining neuroplasticity, promoting neuronal survival, and modulating synaptic function [68]. Their diminution may result in neurodevelopmental abnormalities and persistent neuronal dysfunction, potentially contributing to the chronicity of schizophrenia [15]. Interestingly, studies have indicated that antipsychotic treatment can partially restore BDNF levels [69], while the combination of aripiprazole and NGF-β has been shown to enhance cognitive function in mouse models [70]. These observations highlight the potential of NGF-β and BDNF as biomarkers for chronic schizophrenia, warranting further investigation.

This study had several limitations. First, as a cross-sectional study, it precluded the establishment of causal relationships or the observation of temporal trends. Second, as the active form of vitamin D, 1,25(OH)₂D exbibits variable reference ranges across laboratories and populations. Its levels are influenced by multiple factors, including physical activity, nutrition, calcium homeostasis, parathyroid hormone levels, and renal function, which potentially render it a less reliable indicator of vitamin D status compared to 25(OH)D. Similarly, serum NGF-β and BDNF levels are subject to similar variability and influencing factors. Third, we measured 1,25(OH)₂D, NGF-β, and BDNF levels only in peripheral blood. Given the presence of the blood-brain barrier, discrepancies may exist between peripheral and central nervous system levels of these neurotrophic factors, necessitating further investigation. Finally, although our findings indicate significant associations between neurotrophic factors and chronic schizophrenia, their predictive utility as biomarkers necessitates further validation, especially in light of the potential impact of long-term antipsychotic treatment in chronic patients. Future longitudinal studies involving drug-naïve first-episode patients and ultra-high-risk populations are essential to further elucidate their role as potential biomarkers and to better characterize the confounding effects of antipsychotic medications.

## Conclusions

In conclusion, this study demonstrated significant associations between altered NGF-β and BDNF levels and chronic schizophrenia. The correlations between NGF-β levels and negative symptoms, as well as 1,25(OH)₂D levels, along with the association between BDNF and language deficits, highlighted the significant role of these neurotrophic factors in the pathophysiology of schizophrenia. These findings further enhanced our understanding of the pathophysiological mechanisms underlying chronic schizophrenia. Future studies should focus on two key directions. The first involves exploring the dynamic changes in neurotrophic factors across different stages of schizophrenia and their associations with clinical symptoms and cognitive function. The second entails extending the investigation to female participants to elucidate sex-specific variations in these biological markers, given the well-documented gender differences in symptom presentation and disease progression.

## Data Availability

The data supporting the results of this study are available upon request from the corresponding author.

## Abbreviations

1,25(OH)₂D: 1,25-dihydroxyvitamin D
25(OH)D: 25-hydroxyvitamin D
ANCOVA: Analysis of covariance
BDNF: Brain-derived neurotrophic factor
BMI: Body mass index
DSM-IV: Diagnostic and Statistical Manual of Mental Disorders, Fourth Edition
HC: Healthy controls
IQR: Interquartile range
NGF-β: Nerve growth factor-β
PANSS: Positive and Negative Syndrome Scale
RBANS: Repeatable Battery for the Assessment of Neuropsychological Status
